# Analysis of potential key genes in very early hepatocellular carcinoma

**DOI:** 10.1186/s12957-019-1616-6

**Published:** 2019-05-01

**Authors:** Min Wu, Zhaobo Liu, Xin Li, Aiying Zhang, Dongdong Lin, Ning Li

**Affiliations:** 1 Department of General Surgery, Beijing Youan Hospital, Capital Medical University, 8 Xitoutiao, Youanmenwai, Feng-tai District, Beijing, 100069 China; 2 Beijing Institute of Hepatology, Beijing YouAn Hospital, Capital Medical University, 8 Xitoutiao, Youanmenwai, Feng-tai District, Beijing, 100069 China

**Keywords:** Bioinformatics analysis, Protein–protein interaction network, Differentially expressed genes, Hepatocellular carcinoma

## Abstract

**Background:**

Hepatocellular carcinoma (HCC) is the major pathological type of primary liver cancer, one of the leading causes of cancer death worldwide. In addition, the long-term survival rates of HCC still remain low. Therefore, we attempted to identify the potential key genes in the occurrence of HCC by comparing the expression profiles of very early HCC tissue samples with that of chronic cirrhotic tissue samples by integrating the bioinformatics analysis in this study.

**Methods:**

Gene expression profiles of 19 very early HCC and 19 cirrhotic tissue samples were selected from GSE63898. Differentially expressed genes (DEGs) were also identified by using online tool GEO2R. Furthermore, the GO and KEGG enrichment analysis of the DGEs were conducted on DAVID datasets. Then a protein–protein interaction (PPI) network was constructed and the modules were analyzed based on STRING database and Cytoscape software. The hub genes were screened by applying the cytoHubba plugin and then analyzed with the Kaplan Meier plotter.

**Results:**

A total of 118 DEGs were identified between very early HCC and cirrhotic tissue samples. These DGEs were strongly associated with several biological processes, such as negative regulation of growth and p53 signaling pathway. A PPI network was constructed and top eight hub genes, including CDKN3, CDK1, CCNB1, TOP2A, CCNA2, CCNB2, PRC1, and RRM2, were determined. High expressions of CDK1, CCNB1, TOP2A, CCNA2, PRC1, RRM2, CDKN3, and CCNB2 were associated with poorer overall survivals (OS) in HCC patients.

**Conclusion:**

We had compared the expression profiles between the very early HCC and cirrhotic tissue samples by using bioinformatics analysis tools, which might help us better to understand the molecular mechanism of the initiation of HCC and even to find novel targets for HCC therapy.

## Introduction

Primary liver cancer remains one of the leading causes of cancer death and is a major public health problem all over the world. It was reported that the incidence rate of primary liver cancer continued to increase, and there were 42,220 estimated new primary liver cancer cases yearly in the USA [1]. However, the incidence rate of liver cancer was predicted to decrease in most Asian countries, except for Thailand [2]. Hepatocellular carcinoma (HCC) is the major pathological type of primary liver cancer. The mortality and incidence rates of HCC remain high in Southeast Asia and Africa, where the infection of hepatitis B virus is endemic [3, 4]. Most cases of HCC in these areas are secondary to chronic liver cirrhosis. In contrast, the HCC incidence rate is relatively low in Europe, Australia, and North America, where the major etiologies are chronic alcoholism, iron storage disorder and exposure to aflatoxin [5].

Although the remarkable improvements are achieved in the treatment of HCC such as liver transplantation, radical surgical resection, and interventional therapy [6–8], the long-term survival rates of HCC still remain low worldwide. One of the major reasons is that most patients with HCC were diagnosed at advanced stages. It is crucial to find out new therapeutic targets and novel diagnostic biomarkers for the early diagnosis and timely treatment of HCC. Therefore, it is still urgent to further explore the exact molecular mechanisms of the development, progression, invasion, and metastasis of HCC.

An increasing number of studies have revealed that the initiation of HCC was a complicated process that associated with multiple cellular pathways and numerous genes alterations [9–11]. In addition, Gene Expression Omnibus (GEO) is a public functional genomics data repository that offers us an opportunity to mine the gene expression profiles of all kinds of cancers. Therefore, in this study, we tried to identify and evaluate the clinical values of the potential key genes in the occurrence of HCC by comparing the expression profiles of very early HCC tissue with that of chronic cirrhotic tissue samples by integrating the bioinformatics analysis.

## Material and methods

### Gene expression data of HCC and cirrhotic tissue samples

Gene chip data of 19 very early HCC and 19 cirrhotic tissue samples, which were selected from GSE63898 gene expression profile, was downloaded from GEO. The dataset was provided by Augusto Villanueva, et al and performed on the Affymetrix Human Genome U219 Array [12]. To better illustrate the mechanism of hepatocarcinogenesis, only HCC tissue samples with Barcelona Clinic Liver Cancer (BCLC) stage 0 were selected in this analysis. The basic information of the patients selected in this study was summarized in Table 1 (to reviewer).

**Table 1:** The clinical information of the patients selected

Variable	HCC	LC
Total number	19	19
Median age, years	64	68
Gender		
Female	6	5
Male	13	14
Tumor stage		
Pre-cancer	0	19
BLCL 0	19	0

*HCC* hepatocellular carcinoma; *LC* liver cirrhosis; *BLCL* Barcelona Clinic Liver Cancer

### Identification of differentially expressed genes

Differentially expressed genes (DEGs) between very early HCC and cirrhotic tissue samples were identified by using the Limma package on R language software. DEGs were considered when an adjusted *P* < 0.05 and a |log_2_ fold change|>2. The adjusted *P* values, by employing Benjamini and Hochberg false discovery rate, were aimed to correct the occurrence of false positive results.

### Functional annotation of DEGs

In this study, we used the Database for Annotation, Visualization and Integrated Discovery (DAVID) version 6.8 programs for further functional and pathways analysis of the DEGs. The DAVID analyses of the DEGs mainly contained gene ontology (GO) function analysis and Kyoto encyclopedia of genes and genomes (KEGG) pathways analysis. GO analysis could be further divided into three aspects, namely, biological processes (BP), cellular components (CC), and molecular functions (MF). The cutoff criterion for significant results was a *P* value < 0.05.

### Construction of PPI network and screening of hub genes

To display the interactions and functions of the DEGs, a protein–protein interaction (PPI) network was constructed based on the database of Search Tool for the Retrieval of Interacting Genes/Proteins (STRING) in this study. All the parameters were set as defaults [13]. We applied Cytoscape 3.6.1 software to more intuitively visualize the constructed PPI network. The proteins were represented by nodes, and the protein-interactions were represented by edges in the PPI network. The molecular complex detection (MCODE) plug-in was applied to analyze the modules in the PPI networks, with the default parameters (node score = 0.2, K-core≧2, and max depth = 100). To screen the potential hub genes that may be involved in the hepatocarcinogenesis, we applied the CytoHubba, a Cytoscape plug-in, and the maximal clique centrality algorithm.

To explore the potential clinical significance of the hub genes in HCC, we searched the Kaplan-Meier plotter, which is capable to assess the effect of 54,675 genes on survival using 18,674 cancer samples [14]. The analysis results of overall survivals (OS) with hub genes’ alterations in HCC were figured with log-rank *P* values.

## Results

### DEGs between early HCC and cirrhotic

A total of 200 probe IDs were identified to be differently expressed between very early HCC and cirrhotic tissue samples with the cutoff of adjusted *P* < 0.05 and |log_2_FC|>2. After matching the differed probe IDs with gene symbols from the Affymetrix dataset, 118 genes could be considered as DEGs finally.

### Functional enrichment analyses of DEGs

**Fig. 1:**
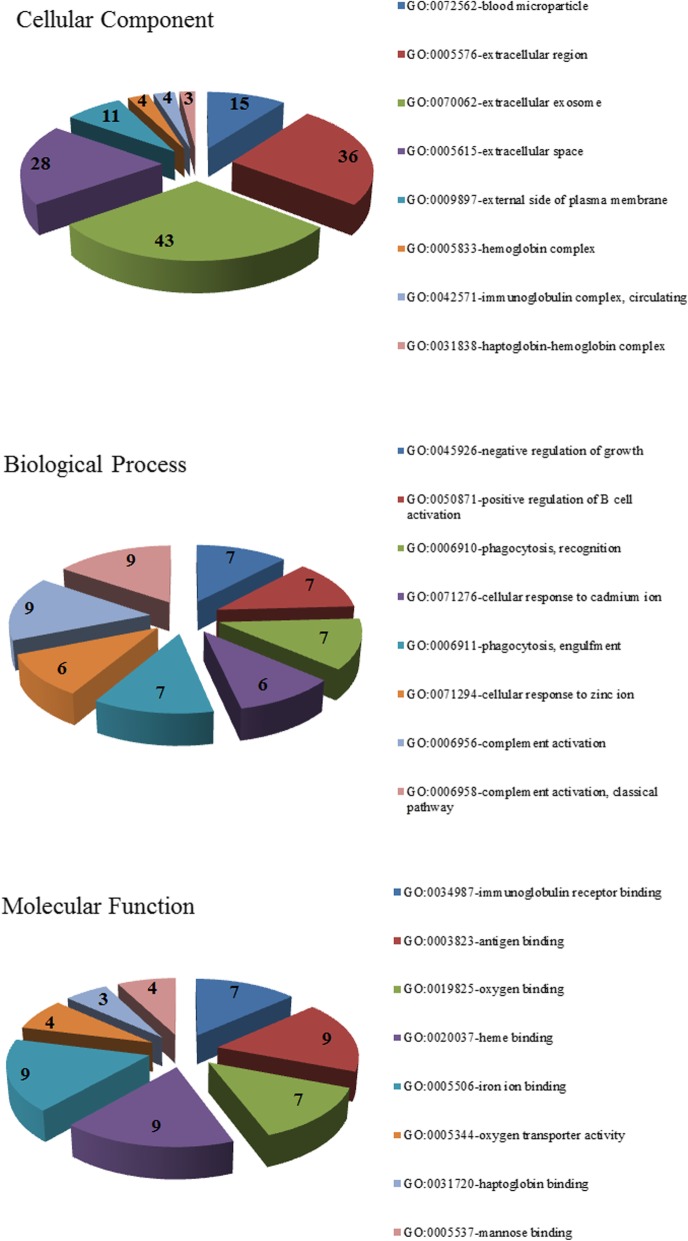
Gene ontology (GO) analysis of differentially expressed genes in very early hepatocellular carcinoma tissue samples

To further understand the functions of the 118 identified DGEs, the GO and KEGG enrichment analyses of the DGEs were conducted and performed in DAVID datasets. The results of GO analysis revealed that the DGEs were mainly involved in 91 biological process terms, such as negative regulation of growth, positive regulation of B cell activation, phagocytosis and recognition, cellular response to cadmium ion, phagocytosis, and engulfment. For the category of cellular component, the DGEs were significantly associated with 20 terms, including blood microparticle, extracellular region, extracellular exosome, extracellular space, and external side of the plasma membrane. Moreover, for the category molecular function, the DGEs were significantly enriched with 27 terms, such as immunoglobulin receptor binding, antigen binding oxygen binding, heme binding, iron ion binding. The top eight terms in each GO category were illustrated in Fig. 1. In addition, the KEGG pathways were significantly enriched for the p53 signaling pathway, chemical carcinogenesis, as listed in Table 2.

**Table 2:** Kyoto Encyclopedia of Genes and Genomes (KEGG) pathway enrichment analyses of differentially expressed genes in very early hepatocellular carcinoma tissue samples

Term	Count	*P* value	Genes
Mineral absorption	6	4.71E-05	MT1M, MT1A, MT1H, MT1X, MT1G, MT1F
p53 signaling pathway	6	2.88E-04	CDK1, CDKN2A, RRM2, IGF1, THBS1, IGFBP3
African trypanosomiasis	4	0.002958176	IDO2, HBA2, HBA1, HBB
Tryptophan metabolism	4	0.005127472	ALDH1B1, IDO2, CYP1A2, INMT
Chemical carcinogenesis	5	0.005272955	CYP3A4, NAT2, HSD11B1, ADH1B, CYP1A2
Malaria	4	0.009039916	HBA2, HBA1, THBS1, HBB
Phagosome	6	0.010951526	MRC1, MARCO, HLA-DRB4, COLEC11, THBS1, CLEC4M
Steroid hormone biosynthesis	4	0.014316303	CYP3A4, HSD11B1, SRD5A2, CYP1A2
Metabolism of xenobiotics by cytochrome P450	4	0.027242121	CYP3A4, HSD11B1, ADH1B, CYP1A2
Salmonella infection	4	0.036531167	FOS, CCL4L1, CCL4L2, CCL4

### Construction of PPI network and analyses of modules

**Fig. 2:**
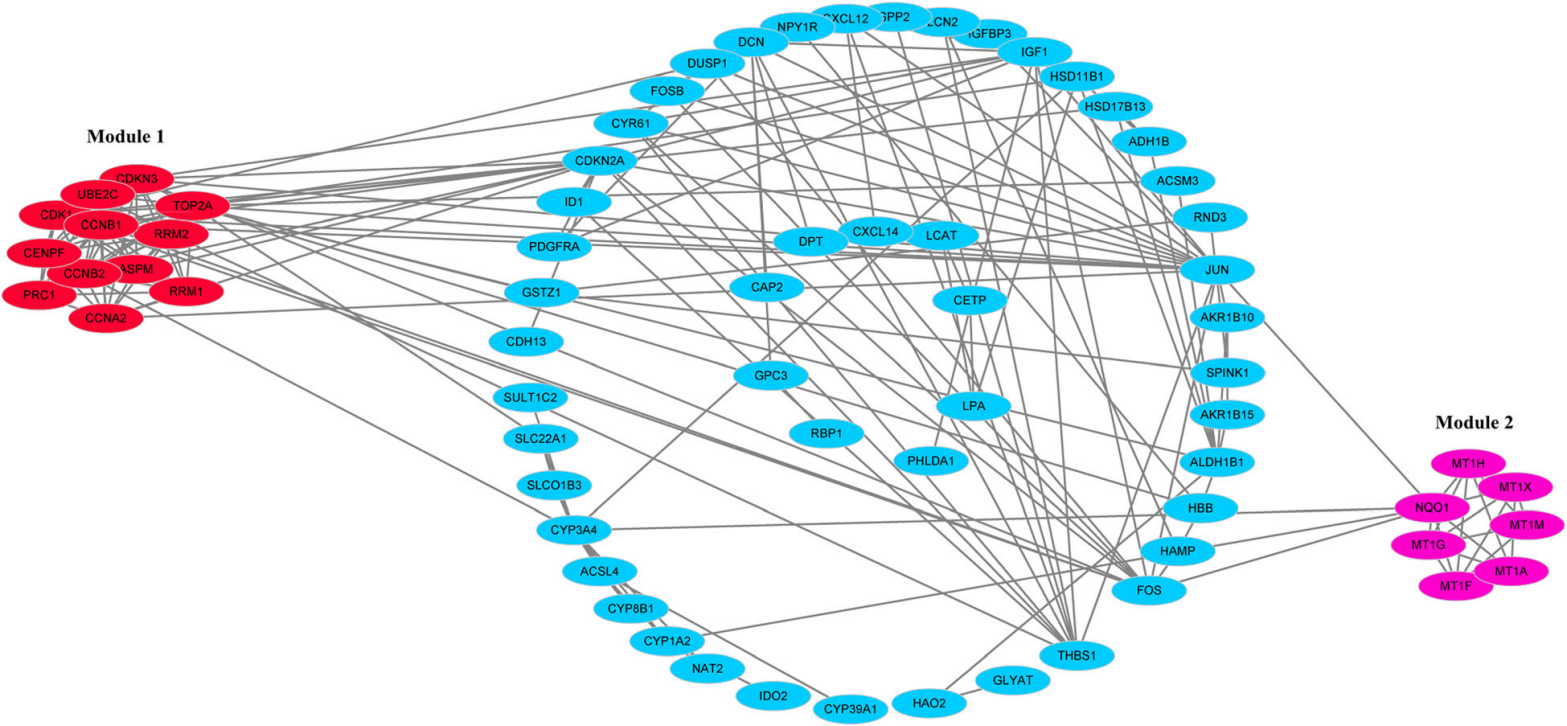
Protein–protein interaction network of the differentially expressed genes in very early hepatocellular carcinoma

**Fig. 3:**
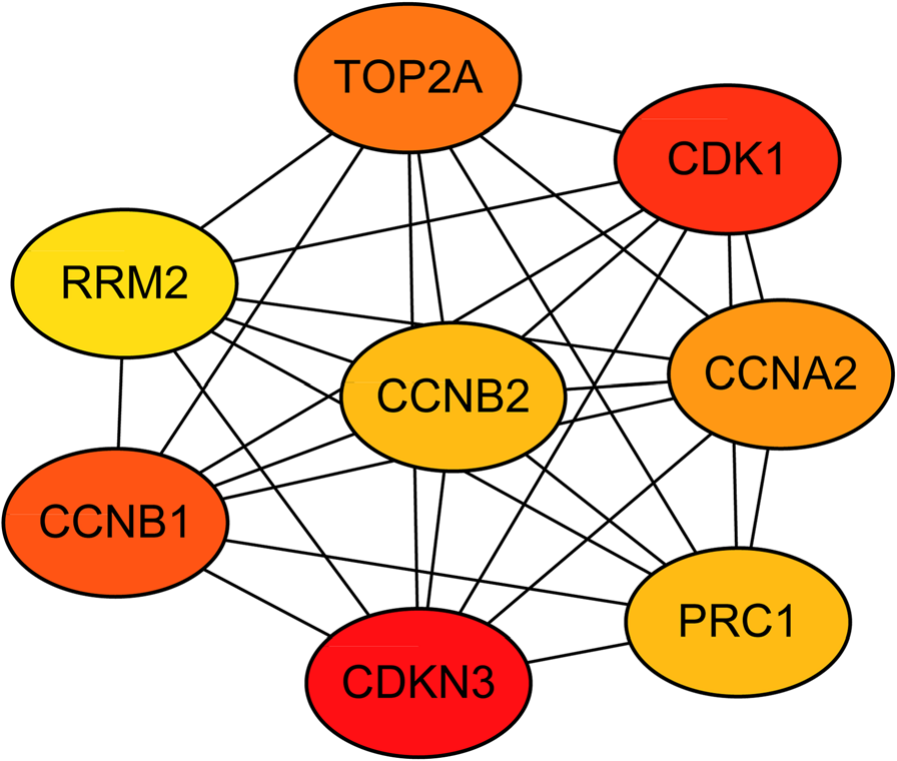
Hub genes screened from protein–protein interaction network

To investigate the protein–protein interactions between the DGEs, a PPI network was constructed based on the STRING database and was visualized by Cytoscape (Fig. 2). The PPI network consisted of 118 nodes and 203 edges. The average node degree of PPI network was 3.44 and the local clustering coefficient was 0.442. The top two modules were chosen after analyzing the entire PPI network by employing the MCODE plug-in (Fig. 2). The hub genes were determined from the PPI network by using the cytoHubba plugin. The top eight hub genes were sequentially listed as follows: CDKN3 (cyclin-dependent kinase inhibitor 3), CDK1 (cyclin-dependent kinase 1), CCNB1 (cyclin B1), TOP2A (topoisomerase (DNA) II alpha), CCNA2 (cyclin A2), CCNB2 (cyclin B2), PRC1 (protein regulator of cytokinesis 1), and RRM2 (ribonucleotide reductase M2) (Fig. 3). All of these hub genes were found to be overexpressed in the very early HCC and included in module 1. Meanwhile, two of these hub genes, including CDK1 and RRM2, were significantly enriched in the p53 signaling pathway.

### Exploring the prognostic value of the hub genes

**Fig. 4:**
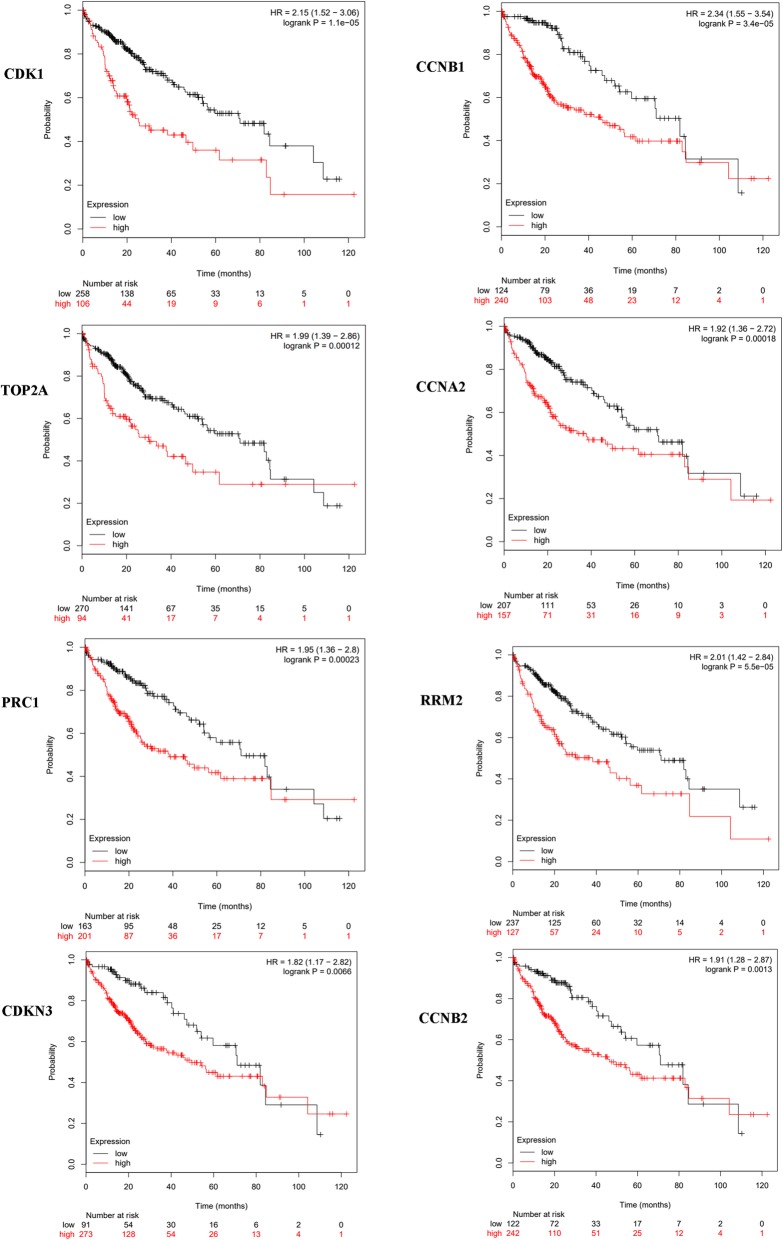
The prognostic value of the hub genes in hepatocellular carcinoma

To further analyze the prognostic value of the identified hub genes, the OS associated with hub genes was analyzed for 364 patients with HCC by using the Kaplan-Meier plotter. High expressions of CDK1 (*P* = 1.1e-5), CCNB1 (*P* = 3.4e-5), TOP2A (*P* = 0.0001), CCNA2 (*P* = 0.0002), PRC1 (*P* = 0.0002), RRM2 (*P* = 5.5e-5), CDKN3 (*P* = 0.0066), and CCNB2 (*P* = 0.0013) were associated with poor OS in HCC patients (Fig. 4).

## Discussion

Although the initiation and progression of HCC had been investigated increasingly in recent years, the exact molecular mechanism of hepatocarcinogenesis is still unclear. In addition, the mortality of HCC remains high worldwide because of the limited therapeutic strategies. Thus, it is particularly urgent to dig deeper to find out the exact mechanisms underlying the development of HCC. Herein, bioinformatics analysis of gene expression profiles obtained from HCC may help us better to understand the molecular mechanism of tumor formation and even to find novel targets for cancer therapy. Bioinformatics analysis of HCC had been investigated in previous studies [15–17]. However, these studies were mainly focused on comparing the gene expressions between benign liver tissue samples and HCC tissue samples at early or advanced stages. Rare studies devoted to finding out the differences between very early HCC tissue samples and benign liver diseases at the gene level, which might be helpful to understand the tumor formation.

In this study, we identified a total of 118 DGEs between very early HCC and cirrhotic tissue samples chosen from the GSE63898 expression profile datasets. GO and KEGG enrichment analyses of these DGEs were also performed in DAVID datasets. The results show that the identified DGEs were closely associated with several biological processes and components, such as negative regulation of growth, extracellular region, and immunoglobulin receptor binding. Moreover, these DGEs were significantly enriched for the p53 signaling pathway, chemical carcinogenesis. Furthermore, to analyze the interactional relationships between the DGEs, a PPI network was constructed and modules analysis was done.

We had also screened out eight hub genes including CDKN3, CDK1, CCNB1, TOP2A, CCNA2, CCNB2, PRC1, and RRM2. These hub genes had been investigated extensively in the previous researches. CDKN3, also known as KAP, CDI1, CIP2, and KAP1, encodes a dual specificity protein phosphatase that had reported to dephosphorylate CDK2. CDKN3 had been overexpressed frequently in several types of cancers, such as breast cancer, prostate cancer, and HCC [18–20]. It had been reported that the overexpression of CDKN3 was correlated with the poor survival in cancer patients [18]. In this study, we found that the overexpression of CDKN3 also had significantly effect on the overall survival of HCC patients. CDK1, a member of the Ser/Thr protein kinase family, plays an essential role in the G1/S and G2/M phase transitions of eukaryotic cell cycle by interacting with CCNB1 [21]. In gastric carcinoma, high expression of CDK1 had found to lead to poor prognosis and correlated inversely with p27 expression [22]. The overexpression of CDK1 was also found to be directly associated with portal invasion, high alpha-fetoprotein level, and poor prognosis in HCC [23], which was in agreement with the results in this study.

TOP2A, encoding DNA topoisomerase II alpha, is involved in processes such as chromatid separation, cell cycle progression and the relief of torsional stress. TOP2A was reported to be frequently co-amplified with HER-2 and then reduce the clinical outcome in urinary bladder cancer and breast cancer [24, 25]. Moreover, TOP2A was also found to be significantly overexpressed and linked with poor prognosis in HCC in this study, which was in conformity with results of previous studies [26, 27]. CCNA2 protein is functioned as a regulator of the cell cycle by activating cyclin-dependent kinases. The expression of CCNA2 was driven by E2Fs [28]. The aberrant expression of CCNA2 could be detected and closely related to reduced survival in HCC and breast cancer [28, 29]. CCNB2 is also a member of the cyclin family, which are core components for cell cycle control. CCNB2 was found to be overexpressed and then result in poor prognosis in non-small-cell lung cancer and invasive breast carcinoma [30, 31].

PRC1 is necessary for microtubules organization and is a substrate of several cyclin-dependent kinases. PRC1 was reported to involve in Wnt/β-catenin signaling pathway and then promote cancer proliferation and tumorigenesis in kinds of cancers, such as HCC and lung adenocarcinoma [32, 33]. RRM2 plays a critical role in converting ribonucleotides to deoxynucleotides in DNA synthesis. The elevated expression of RRM2 may lead to the poor prognosis for patients with non-small cell lung cancer by stabilization of Bcl-2, which is a critical regulator of apoptosis [34]. In addition, it had been shown RRM2 might be a novel therapeutic target in variety of cancers, such as breast cancer, non-small cell lung cancer, and bladder cancer [34–37].

## Conclusion

In summary, we attempted to find out the differences between the very early HCC tissue samples and cirrhotic tissue samples at gene level by using bioinformatics analysis tools. A total of eight key genes in the initiation of HCC were identified and their prognostic values were explored based on Kaplan-Meier plotter. However, further experimental verification is necessary to confirm the results in this study.

## Abbreviations

CCNA2: Cyclin A2
CCNB1: Cyclin B1
CCNB2: Cyclin B2
CDK1: Cyclin-dependent kinase 1
CDKN3: Cyclin-dependent kinase inhibitor 3
DAVID: Database for Annotation, Visualization and Integrated Discovery
DEGs: Differentially expressed genes
GEO: Gene Expression Omnibus
GO: Gene ontology
HCC: Hepatocellular carcinoma
KEGG: Kyoto Encyclopedia of Genes and Genomes
OS: Overall survivals
PPI: Protein–protein interaction
PRC1: Protein regulator of cytokinesis 1
RRM2: Ribonucleotide reductase M2
STRING: Search Tool for the Retrieval of Interacting Genes/Proteins
TOP2A: Topoisomerase (DNA) II alpha
